# Learning to Discern Images Modifies Neural Activity

**DOI:** 10.1371/journal.pbio.0020062

**Published:** 2004-02-17

## Abstract

xx

The primate brain processes a remarkably diverse array of visual cues to recognize objects in dynamic settings crammed with unfamiliar objects. Not surprisingly, repeated viewing aids recognition, but how the brain orchestrates this experience-driven improvement is unclear. Visual input to the brain travels from the eye to the primary visual cortex (V1), at the back of the brain. From there, signals are sent to nearby extrastriate cortical areas, which process “early” visual cues. Both the “lower level” extrastriate cortex and “higher level” inferior temporal (IT) cortex are important for object recognition in primates. In monkeys and humans, lesions in the IT cortex severely affect the ability to recognize objects.

In these higher-level cortical regions, neurons carry more information about an object after subjects learn to recognize that object. This modified neural activity is thought to reflect internal representations of specific aspects of the learned task—such as learned recognition of three-dimensional objects—and these representations often remain stable even though certain features of the visual stimulus—such as size or image degradation—change. With recent evidence suggesting that lower level brain regions like the primary visual cortex are also capable of learning-related modifications, it appears that both early and higher brain areas of the “ventral visual stream” benefit from learning. It is not clear, however, how learning modifies these discrete brain regions to coordinate this processing.

By training monkeys to recognize degraded images, Gregor Rainer, Han Lee, and Nikos Logothetis of the Max Planck Institute for Biological Cybernetics in Germany have identified a subset of neurons that compensate for indistinct visual inputs by coordinating disparate regions in the brain. The monkeys' improved performance, they propose, stems from the informational enrichment of a subset of lower level neurons. Along with an increase in learning-induced firing activity, V4 neurons—extrastriate cortical neurons associated with detecting visual input of intermediate complexity—encode more information about relevant details to resolve indeterminate visual cues. V4 neurons likely interact with higher cortical levels to help the monkeys interpret the degraded indeterminate images as something recognizable.

The researchers presented the monkeys with different “natural” images, including pictures of birds and humans, then subjected the images to different levels of “stimulus degradation”—making them harder to read by adding varying amounts of visual noise. Using this approach, the researchers could record the activity of the V4 neurons as the monkeys were presented with the different images. The monkeys viewed a sample image and then signaled whether a second image, presented after a brief delay, was a match or not. When Rainer et al. analyzed the activity of the V4 neurons associated with the different images, they found there was no significant change in the activity or information conveyed by V4 neurons associated with novel or undegraded familiar images. On the other hand, learning not only significantly improved the monkeys' ability to recognize degraded stimuli but also increased both the activity and informational encoding of the V4 neurons.

But how did individual V4 neurons facilitate this enhanced ability to recognize degraded stimuli? After identifying a subset of neurons that showed enriched neural activity in response to degraded or indeterminate stimuli, the researchers studied the monkeys' eye movements to determine any behaviors that might explain why monkeys performed better with familiar degraded stimuli. They mapped the monkeys' eye movements while allowing them to freely view the different familiar and novel images—but this time with just two coherence levels (undegraded and 45% coherent). There was substantially more overlap, in terms of where the monkeys looked for the 45% and 100% coherent images after learning. This suggests that monkeys learned to focus their attention on particular salient features, and were thus better able to identify degraded versions of these images.

Neurons in the V4 area appear to be recruited to distinguish the relevant visual signal from the visual noise, and thus play a critical role in resolving indeterminate stimuli when salient features are present. These results, together with previous studies showing the sensitivity of prefrontal cortex neurons to novel stimuli, indicate that the prefrontal cortex processes novel stimuli while the V4-rich extrastriate visual areas convey details about hard to decipher images. It may be that as the V4 neurons refine their competence through learning, they also support the ability of the prefrontal cortex to process different but similar visual cues. Vision is a dynamic process, Rainer et al. conclude, characterized by ongoing interactions between stimulus-driven brain regions and feedback from higher-order cognitive regions.

**Figure:**
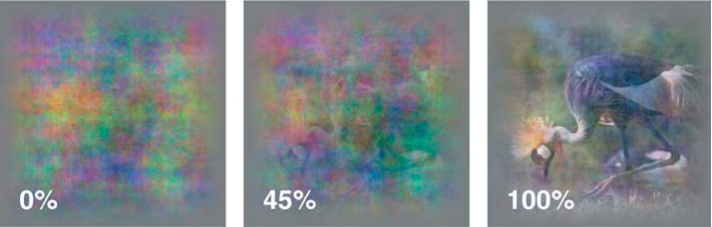
Monkeys can learn to recognize degraded images

